# Location and Language Independent Fake Rumor Detection Through Epidemiological and Structural Graph Analysis of Social Connections

**DOI:** 10.3389/frai.2022.734347

**Published:** 2022-04-27

**Authors:** Dimitrios Serpanos, Georgios Xenos, Billy Tsouvalas

**Affiliations:** ^1^Computer Technology Institute and Press Diophantus, Patras, Greece; ^2^Department of Electrical and Computer Engineering, University of Patras, Patras, Greece; ^3^Industrial Systems Institute/ATHENA, Patras, Greece; ^4^Computer Science Department, Virginia Commonwealth University, Richmond, VA, United States

**Keywords:** misinformation, rumor propagation, rumor classification, epidemiological models, graph-based detection

## Abstract

Detection and identification of misinformation and fake news is a complex problem that intersects several disciplines, ranging from sociology to computer science and mathematics. In this work, we focus on social media analyzing characteristics that are independent of the text language (language-independent) and social context (location-independent) and common to most social media, not only Twitter as mostly analyzed in the literature. Specifically, we analyze temporal and structural characteristics of information flow in the social networks and we evaluate the importance and effect of two different types of features in the detection process of fake rumors. Specifically, we extract epidemiological features exploiting epidemiological models for spreading false rumors; furthermore, we extract graph-based features from the graph structure of the information cascade of the social graph. Using these features, we evaluate them for fake rumor detection with 3 configurations: (i) using only epidemiological features, (ii) using only graph-based features, and (iii) using the combination of epidemiological and graph-based features. Evaluation is performed with a Gradient Boosting classifier on two benchmark fake rumor detection datasets. Our results demonstrate that epidemiological models fit rumor propagation well, while graph-based features lead to more effective classification of rumors; the combination of epidemiological and graph-based features leads to improved performance.

## Introduction

Information diffusion describes the way in which information is disseminated through a network (Rogers et al., 2014). Although the original meaning of the process encompasses interpersonal communication channels (Katz and Lazarsfeld, 2017), given the increasing popularity of social media in the last two decades, information diffusion, and especially rumor propagation nowadays, tends to be linked with the dissemination of information over specific digital social networking platforms, such as Twitter. The Diffusion of Innovations (DOI) theory (Rogers et al., 2014) states that information diffusion is not solely determined by attributes of the statement or the novelty introduced, but it is also integrally related to the communication channel properties, i.e. the individuals involved or other network characteristics. Considering the accelerated pace of information diffusion in the digital age, it becomes readily apparent that rumor propagation and fake news dissemination is developing into a critically complex issue, with many and convoluted parameters.

Three techniques have been mostly employed in tackling this issue: structural analysis, temporal analysis, and content-based techniques. Structural analysis refers to the analysis of information cascades and the communication channel properties, whereas temporal analysis takes into account the transitional characteristics of information diffusion and aims to arrive at conclusions by evaluating the diffusion activity based on time. Content analysis is the most commonly used technique; by capitalizing on Natural Language Processing (NLP) advances in the last decade, it manages to offer contextual characteristics to the information diffusion problem. An essential aspect of these techniques is their ability to generalize for claims, i.e. whether the extracted results are appropriate to make a generalized claim. Content analysis provides some clear comparative advantages over the other two techniques, but it is location-specific and dependent on the *ad hoc* regional and social context (Siwakoti et al., 2021); thus, it does not generalize easily for several social media in various countries. On the other hand, a structural or temporal analysis of information diffusion offers universal results, which are location-independent and language-independent.

Significant research effort has been spent studying and modeling information diffusion on social media (Jin et al., 2013, 2014; Cannarella and Spechler, 2014; Goel et al., 2016). Fake news propagation and classification have received significant attention in the past few years and have been analyzed with a variety of techniques that include propagation-based, content-based and social context-based characteristics. However, existing work takes into account content information, which makes the analyses dependent on the language and the social context of users. This is also due to the scarcity of relevant datasets, which leads to results based on the few available datasets, mainly Twitter (Ma et al., 2017). In contrast, our work focuses on analysis that does not consider language and social context characteristics, enabling a location and language independent fake rumor detection.

In this paper, we focus on the problem of rumor modeling and extraction. Considering that most literature adopts the term “fake news,” in our work, we adopt the term “rumor” because we differentiate rumors from fake news as follows: we consider that rumors are doubtful statements that cannot be easily verified, while fake news is intentionally fabricated information presented as true. The difference, although subtle, distinguishes the data samples on which our methodology applies, from the generic ones that appear in the literature as samples of misinformation data, in general.

In our work, we perform temporal analysis through epidemiological models over the information diffusion on Twitter and we extract specific temporal features; the choice of Twitter is due to the availability of suitable datasets. Additionally, we analyze the graph structure of the information cascade of the communication channel and we extract graph-based features. Having extracted the features using these two techniques, we proceed to evaluate them in three classification schemes: (i) using only epidemiological features, (ii) using only graph-based features, and (iii) using a combination of epidemiological and graph-based features. More specifically, the contributions of our work are the following:

1. We show that epidemiological models fit rumor propagation data, especially model SEIZ, and we evaluate their performance on fake rumor classification tasks on Twitter datasets.
2. We construct a graph model of the information diffusion and we extract graph-based features of the propagation.
3. We perform binary and multi-class classification using the graph-based features and a combination of graph and epidemiological features, achieving higher performance.
4. We present a location-independent and language independent fake rumor detection method.

The paper is organized as follows. Section Related Work presents prior work. In section A Location-Independent and Language-Independent Approach, we present the epidemiological models and the graph modeling method as well as the employed classification schemes; for the epidemiological models we describe how they are employed in fitting the rumor propagation data and for the graph models how feature extraction is accomplished. Section Experiments and Evaluation presents our evaluation datasets and the results of rumor classification performance using epidemiological features, graph-based features and the combination of both. Section Conclusions presents our conclusions and directions for future work.

## Related Work

Epidemiological models have been used extensively to model information diffusion in complex networks. Such models classify the human population into different compartments and define different transitions between them to simulate the spread of information. The SI (Susceptible-Infected) model was originally proposed in 2001 (Pastor-Satorras and Vespignani, 2021) indicating that epidemiological models can help to describe the propagation of information on scale-free networks. Later another variant, the SIS (Susceptible-Infected-Susceptible) model was introduced (Newman, 2003) and used multiple times (Gross et al., 2006; Jin et al., 2013, 2014); the model allows infected users to return to the Susceptible compartment. Other more complex models have been proposed and used, such as the SEIR (Susceptible-Exposed-Infected-Removed) model (Wang et al., 2014), the S-SEIR model (Xu et al., 2013), where the spread of information is dependent on its value to the user, and the SCIR model (Xuejun, 2015) where a Contacted compartment is added, modeling how followers of a certain user react after he posts an online message. Another approach (Cannarella and Spechler, 2014) considers a modification of the SIR model where the adoption of an idea is considered an infection and its abandonment is considered a recovery. A more widely used model is the SEIZ (Susceptible-Exposed-Infected-Skeptics) model (Bettencourt et al., 2006); this model can fit long term ideas adoption. Recently, the SEIZ was employed to model the spread of fake and real news on Twitter (Jin et al., 2013, 2014). The authors proposed a simple method to classify news as either true or fake using the results of the SEIZ fitting; while the model performs well, the authors have used only a small amount of viral news stories to test their hypothesis.

Another promising and popular research direction related to the propagation of rumors and fake news is the exploitation of the diffusion graphs. Typically, graphs are constructed, representing information diffusion paths, and then features are extracted, which are used either to interpret the propagation, or to perform classification based on a scheme. In the following sections, we discuss how graphs and graph characteristics are employed to address the information diffusion problem. There have been several approaches aiming to provide a qualitative context in the way that information is disseminated. By studying the diffusion patterns in a social network along with the significance of individual nodes (users), one may recognize specific individuals as critically important to the information diffusion (Valente, 1996). Such users, who may be characterized as “opinion leaders,” tend to be the connection between mass media and people in the community (Katz and Lazarsfeld, 2017). Furthermore, they are more important than average individuals in the diffusion of information (Watts and Dodds, 2007) and have been linked with the size and the structural virality of the diffusion (Goel et al., 2016; Meng et al., 2018). Along the same lines of research, efforts have also been directed to understand the clustering patterns of news propagation (González-Bailón and Wang, 2013). Analysis of such clusters has yielded a deeper comprehension of the importance of “brokers,” or users that connect otherwise separated clusters. Such qualitative interpretations of the information diffusion graph structure (nodes and edges) are crucial to understand how diffusion efficiency is related to the network users (Valente and Fujimoto, 2010). Other research efforts, usually directed toward detection or classification schemes, focus more on extracting structural graph features relevant to diffusion. While there are attempts that concentrate on graph characteristics, such as the size of the cascade (graph), the root outgoing degrees, the followers count, or the geo-coordinates (Taxidou and Fischer, 2014), most directions employ a combination of temporal, graph, and/or content analysis (Abulaish et al., 2019; Lu and Li, 2020; Wu et al., 2020; Lotfi et al., 2021). Although most research on information diffusion and rumor propagation contains text and content analysis, such features are location-specific and do not generalize universally (Siwakoti et al., 2021). Based on this, in our work, we employ a framework that consists of a temporal aspect in the form of an epidemiological model, and a graph-based structure in the form of the information cascade of each rumor.

Baseline rumor detection approaches are categorized based on their different feature engineering methods. The categories are (i) user characteristics, (ii) social context and content, and (iii) propagation structure characteristics. Handcrafted features based on user characteristics include such features as the number of followers, number of posts, and relevant information referring to the user of the social media platform (Kwon et al., 2013; Liu et al., 2015). Feature engineering employing social context, content, and linguistic attributes has been proven to yield important results. Such features include word sequences, phrase inquiries, and the relation of specific language with sentiment (Castillo et al., 2011; Yang et al., 2012; Kwon et al., 2013, 2017; Ma et al., 2015, 2016, 2018; Zhao et al., 2015; Liu and Wu, 2018; Yuan et al., 2020; Choi et al., 2021). Features based on the structural characteristics of the propagation have also been an integral part several rumor detection approaches (Ma et al., 2017, 2018; Liu and Wu, 2018). These graph-based features include information referring to the diffusion of a rumor in a social media network.

Several existing methods have explicitly proposed feature engineering, in order to improve the achieved accuracy (Castillo et al., 2011; Yang et al., 2012; Kwon et al., 2013, 2017; Liu and Wu, 2018; Ma et al., 2018). Below, we elaborate further on rumor detection approaches that include feature engineering and we discuss the baseline approaches of the field.

A decision tree classification (DTC) approach using word frequency, message, user, topic, and propagation characteristics to extract features has shown that such combinations allow for promising results (Castillo et al., 2011). Another content-oriented approach includes inquiring for specific phrases in the message (Zhao et al., 2015). The combination of content and user characteristics employing an RBF kernel Support Vector Machine (SVM-RBF) for classification (Yang et al., 2012) has demonstrated that the combination of such features is valid and achieves good results, while Support Vector Machine algorithms have also been used to classify features combining the temporal evolution of the content, along with the relevant social context and the underlying linguistically expressed sentiment with great success (Ma et al., 2015). A content-based approach using language pattern features from user comments and employing a Recurrent Neural Network (RNN) has also delivered important results (Ma et al., 2016), while others have attempted to combine the structure and content, in the form of semantic analysis, to perform the classification tasks (Ma et al., 2018). Along the same lines, content semantics and structural characteristics are the basis for feature engineering for similar attempts (Liu and Wu, 2018). More propagation-based heavy approaches have examined the temporal evolution of the propagation tree and classification takes place using an SVM classifier (Ma et al., 2017). A more holistic approach combines user characteristics, linguistic, and network features in a Random Forest classification scheme (Kwon et al., 2017).

## A Location-Independent and Language-Independent Approach

In this section, we present the specific epidemiological models employed to model rumor propagation in online social media. We define and describe the function of the fitting parameters of these models, and we present the governing formulae for each model. Furthermore, we elaborate on feature engineering, regarding the graph modeling component of our approach, and we present the method that combines the two modeling practices in a rumor classification scheme.

### Epidemiological Models

We use epidemiological models to model the diffusion of rumors and detect whether they are false or not, focusing on the SI and SEIZ models. The SI model is employed for its adaptability to scale-free networks (Pastor-Satorras and Vespignani, 2021), which allows its extension to rumor propagation problems, while the SEIZ model has already been proven to model fake news diffusion effectively (Jin et al., 2013, 2014). Below, we present the model definitions and how they can specifically be used to model rumor diffusion on social networks.

#### SI Model

The SI model classifies the total population of users (N) in two groups, namely the Susceptible (S) and Infected (I) compartments. A user is considered *Infected* if she/he has retweeted the original rumor tweet and *Susceptible* if she/he has not retweeted. Thus, a user stays indefinitely in the Infected state and cannot move back to the Susceptible state. This means that, in the beginning of the spread, the majority of users are in the Susceptible state and, after a sufficient time period, all users will end up in the Infected state. The rate of contact (state change) between the susceptible and infected populations per given unit of time dt is β.

The SI model is described formally by the following ordinary differential equations system:

(1) $$\begin{array}{r} \frac{dS}{dt}\;=\;-\beta SI \end{array}$$

(2) $$\begin{array}{r} \frac{dI}{dt}\;=\;\beta SI \end{array}$$

where *N*(*t*) = *S*(*t*) + *I*(*t*)

#### SEIZ Model

**Figure 1:**
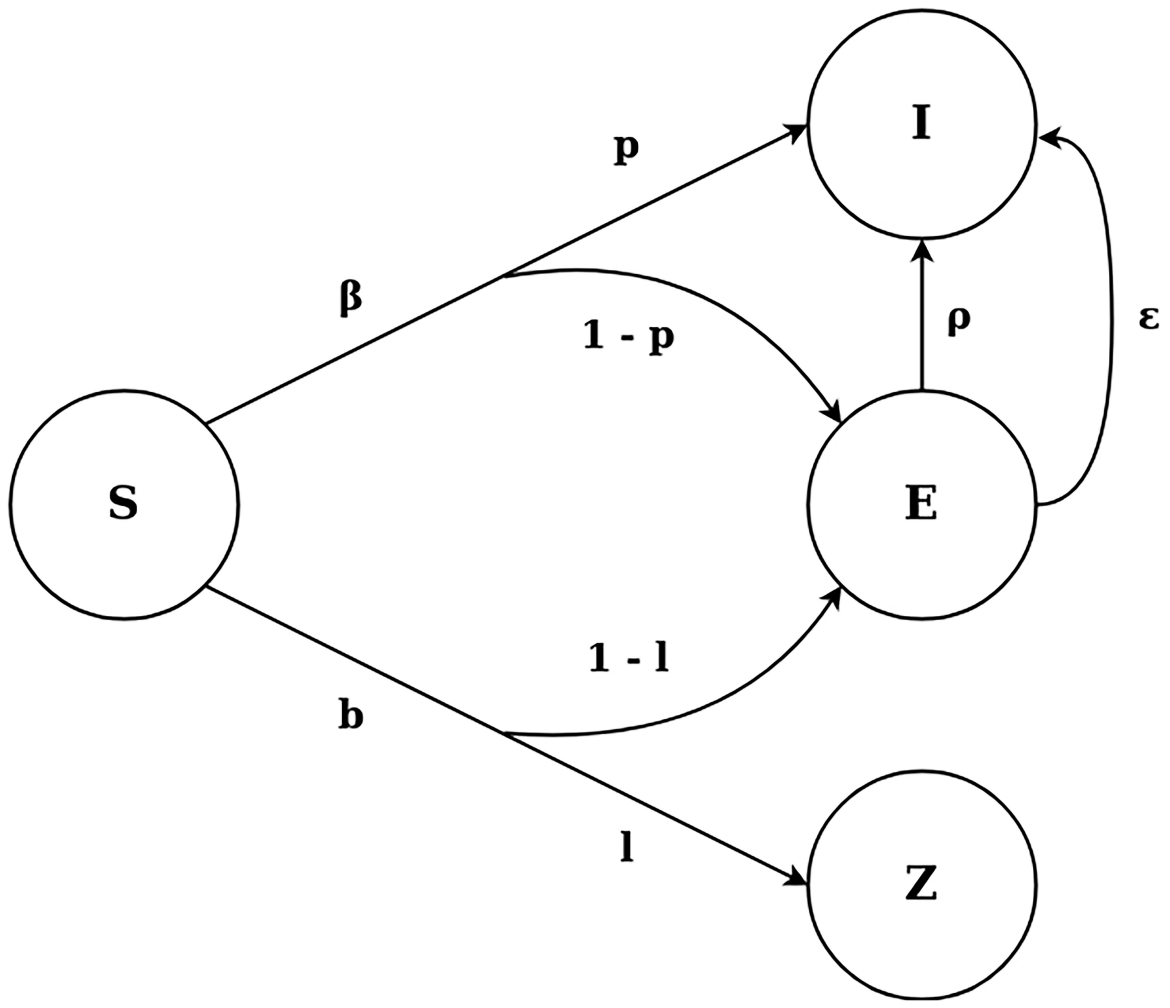
The SEIZ model.

The SEIZ model, as adopted by Jin et al. (2013), is composed of four different compartments, *Susceptible* (S), *Exposed* (E), *Infected* (I) and *Skeptics* (Z) and is shown in Figure 1. The Susceptible state represents users who have not seen the original tweet, while the Exposed state represents the users who have seen the original rumor tweet but take some time before retweeting the original tweet. Infected are considered the users who have retweeted the rumor and Skeptics denote the users who have seen the tweet but have chosen not to retweet it. A Susceptible user can transfer to the Skeptics state with a rate b and probability l or to the Exposed state with probability (1–l). At the same time, a Susceptible user can immediately believe the rumor and move to the Infected state with probability p or to the Exposed state with probability (1–p). Finally, an Exposed user can transfer to the Infected state in 2 different ways: (i) by coming in further contact with an Infected user, with contact rate ρ, or (ii) by adopting the rumor independently with rate ε.

The SEIZ model is defined by a set of different parameters (Jin et al., 2013), as depicted in Figure 1. The contact rates between the different user states quantify how often a user gets in contact with a user of another state (β, b, e for the S-I, S-Z, E-I transitions, respectively). These rates multiplied with the probability of a user changing state when in contact with another user (l, 1-l, p and 1-p for the S-Z, S-E, S-I and S-E transitions, respectively) give the effective rate of users changing states [bl, βρ, b(1-l), β(1-p) for the S-Z, S-I, S-E via Z, S-E via I transitions, respectively]; this effective rate is the rate at which users change states. Finally, an incubation rate (ε for the E-I transition) defines how often a user changes state without getting in contact with any other user.

The SEIZ model is represented by the following ordinary differential equations system (Jin et al., 2013):

(3) $$\begin{array}{r} \frac{dS}{dt}\;=\;-\beta S\frac{I}{N}\;-\;bS\frac{Z}{N} \end{array}$$

(4) $$\begin{array}{r} \frac{dE}{dt}\;=\;\left(1-p\right)\;\beta \;S\frac{I}{N}\;+\left(1-l\right)\;bS\;\frac{Z}{N}\;-\;\rho E\frac{I}{N}\;-\;\epsilon E \end{array}$$

(5) $$\begin{array}{r} \frac{dI}{dt}\;=\;p\beta S\frac{I}{N}\;+\;\rho E\frac{I}{N}\;+\;\epsilon E \end{array}$$

(6) $$\begin{array}{r} \frac{dZ}{dt}\;=\;lbS\frac{Z}{N} \end{array}$$

#### Fitting—Parameter Identification

For each rumor we quantify the different model parameters, in order to use them as features to classify the rumors. To achieve this, we calculate the optimal values for each given model as well as the optimal initial populations of the compartments and the total population (N) for each rumor. For both the SI and the SEIZ models we perform a least squares fitting on the Twitter data. As described earlier, we consider everyone who has retweeted or replied to an original tweet as Infected (I).

**Figure 2:**
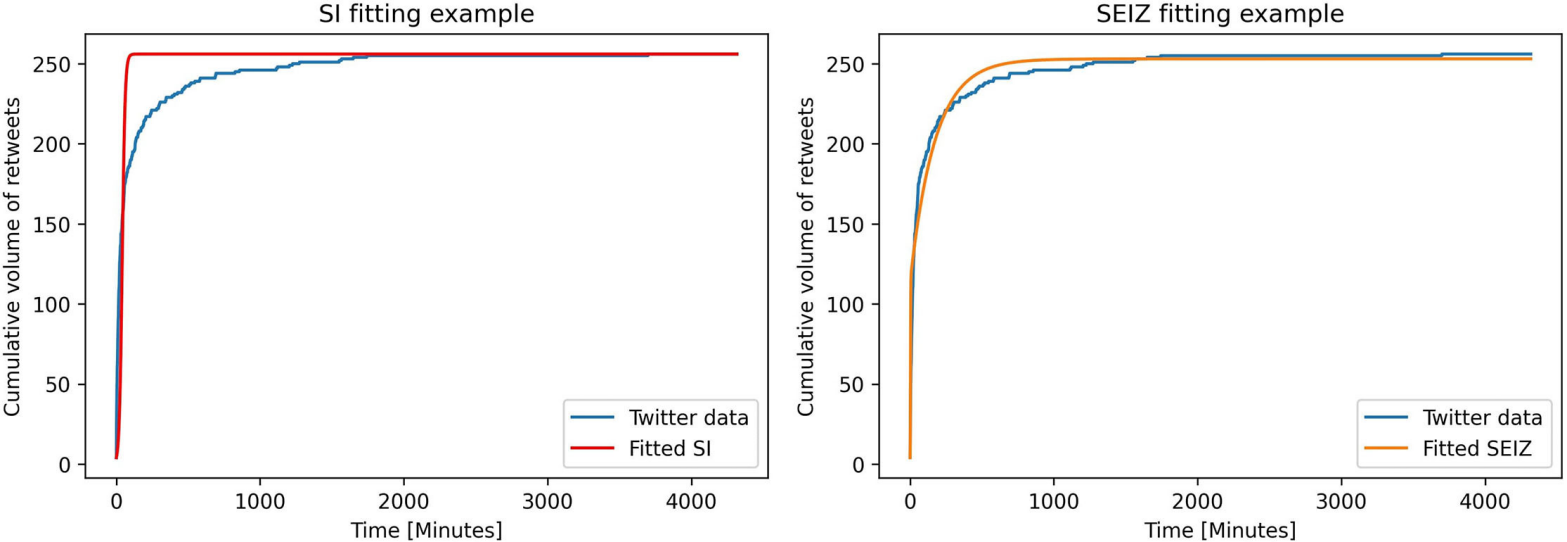
SI and SEIZ fitting examples for the first 72 h of diffusion of a particular rumor.

To fit the epidemiological models on the datasets we first pre-process the raw data to construct sequences that give the cumulative volume of retweets per given time unit (time interval). In our approach we use 1 min time intervals. We also observe that most of the retweets happen early on in the diffusion tree and thus we restrict the fittings of the models only to the first 240 h (or 10 days) of diffusion. More specifically, we perform several fittings using a limit of 48, 72, 120 and 240 h. Finally we fit the models using a time limit of just 4 h to evaluate if the models are able to fit the early diffusion of rumors. Figure 2 presents an example of the abovementioned fitting for both the SI and SEIZ models.

For each fitting we measure the root mean square error (RMSE) between the fitted and the observed cumulative retweets time sequences. We then average the RMSE for each dataset to evaluate and compare the total fitting processes for every dataset and time limit.

### Graph Models

Graph modeling constitutes our second approach; we describe the extraction of graph-based features. Considering that we use Twitter datasets for the evaluation of our methods, the description takes into account the features of the specific datasets, although the method can be applied to graphs that are extracted from datasets originating from alternative social platforms as well. In the case of the Twitter datasets, it is important to note that the datasets are labeled. Each rumor cascade is labeled as True, False, Unverified, or Non-Rumor.

#### Graph Modeling

Considering the dataset characteristics, the directed graph structure is composed of nodes that represent the Twitter users involved in the cascade, and edges that represent the retweeting action performed by the destination user on the message appearing on the source user's timeline. The edges' weights are equal to the absolute time of diffusion. The root user is considered to send the message at time *t* = 0.

For the graph modeling of every cascade in the datasets, we note that the graph edge weights, which represent the time of the retweet, are absolute in value. In case of a negative weight appearing in the data (due to dataset inconsistencies), we re-calibrate the whole cascade by adding an offset value to all diffusion times, following Eq. 7. Furthermore, we consider that diffusion takes place only when a user has retweeted the message. Thus, each destination user appears only once, since a user can retweet a message at most once. We also note that, since we only consider the retweeting action as the edge connection of the graph, a source user cannot be a destination user (retweeting is only one-way). Following these considerations, the resulting rumor propagation graph is acyclic.

(7) $$\begin{array}{r} offset=|(diffusion\text{\_}time{)}_{\min}|\;,where\;(diffusion\text{\_}time{)}_{\min}\;<\;0 \end{array}$$

**Figure 3:**
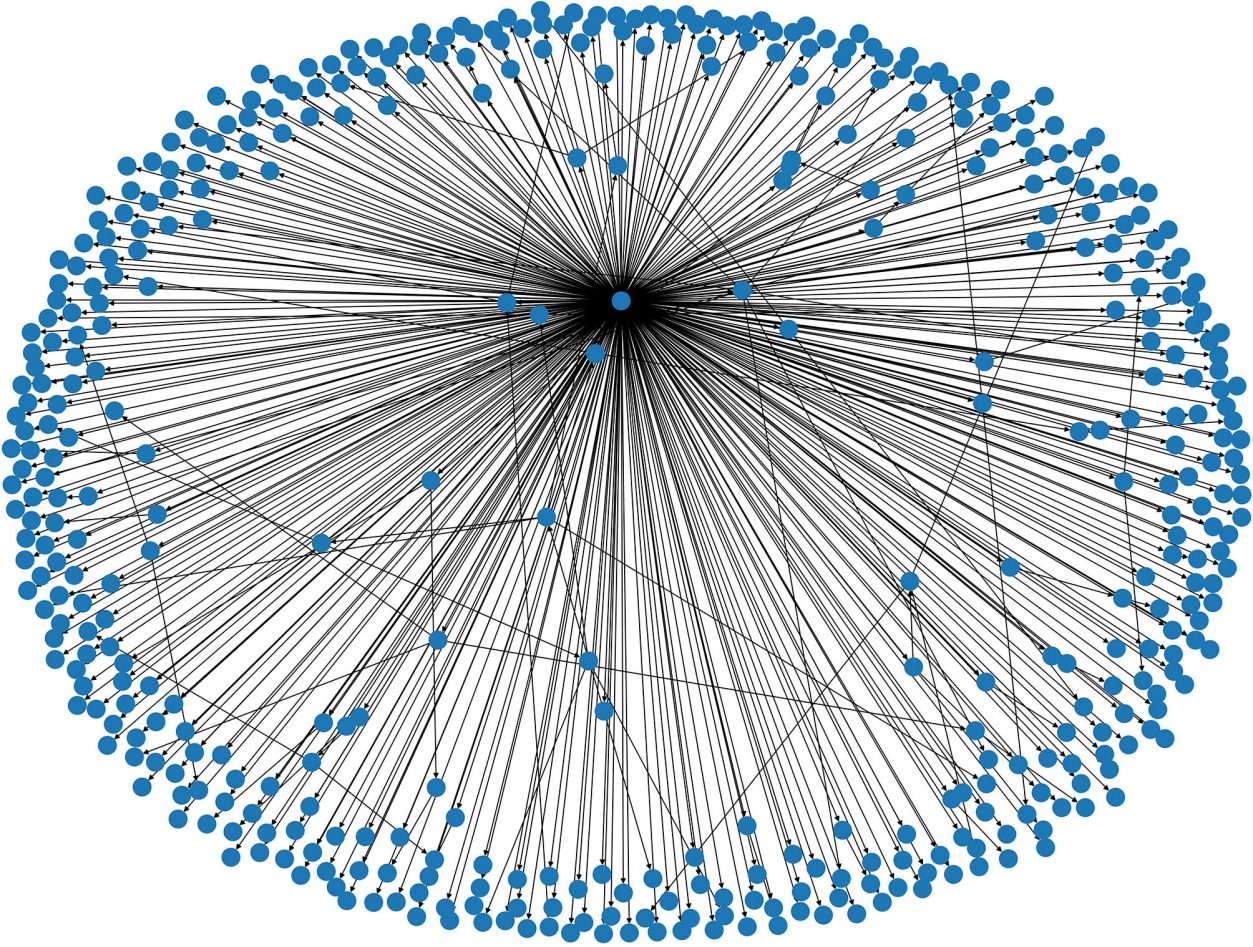
Graph of false story cascade from the Twitter16 dataset.

In Figure 3, we present the graphical representation of a false story cascade from Twitter16, which demonstrates the above characteristics. In this example, the root is clearly the user node with the most outgoing edges.

#### Feature Extraction

Given the constructed graph, we extract the relevant features. For each cascade, the graph-based feature set contains the following:

- Average degree: The average number of outgoing edges for every user.
- Root degree: The total outgoing edges of the root user.
- Structural virality: A graph theoretic metric for measuring the structural diversity of information diffusion. Describes the manner of diffusion; single broadcast or through several cascading individual nodes.
- Closeness centrality: A measure of the nodes' capability to spread information efficiently through the graph. It measures the inverse distance of a node to all other nodes.
- Number of users: The number of unique Twitter users involved in a cascade.
- Max hops: The maximum number of hops (node to node via edge) possible in a cascade.
- Nodes of levels 0 and 1: The number of users at one and two degrees of separation from the root user respectively.
- Baseline average diffusion time for levels 0 and 1: The average diffusion time for users at one and two degrees of separation away from the root user respectively (average calculated by sum of edge weights divided by population involved on level).
- Average diffusion time (averaged over all cascade users) for levels 0 and 1: The average diffusion time for users at one and two degrees of separation away from the root user respectively (average calculated by sum of edge weights divided by population involved in whole cascade).

The last two features are both closely related to the levels of the diffusion. We note that for these features, the calculations are performed with no upper time threshold. However, we also calculate both of them using an upper time threshold, so as to simulate early detection and be relevant to the epidemiological model results. The calculations involve upper limits of 4, 24, 48, and 72 h.

We use the collected features for the calculation of the average diffusion times per level of diffusion for each type of cascade (True, False, Unverified, Non-Rumor). We calculate the average diffusion time per level as follows:

(8) $$\begin{array}{r} avg\;=\;\frac{\sum diffusion\text{\_}time\text{\_}of\text{\_}users\;\in \;level_{i}}{number\text{\_}of\text{\_}users\;\in \;level_{i}}\cdot \\ \frac{number\text{\_}of\text{\_}level_{i}\text{\_}instances}{total\text{\_}number\text{\_}of\text{\_}cascades\text{\_}of\text{\_}same\text{\_}label} \end{array}$$

where the number of level instances refers to the number of cascades that have max hops greater or equal than the specific level.

### Graph and Epidemiological Models Combination

After identifying the appropriate epidemiological models and extracting the relevant features, we perform tests to find a potential correlation between the epidemiological and the graph-based features. It has not been feasible to identify such correlation. Thus, we merge the two feature sets, in order to arrive at higher classification accuracy. The motivation to merge the two feature sets originates from the fact that the epidemiological features represent temporal characteristics of the rumor diffusion, whereas the graph-based features represent structural characteristics of the diffusion cascade of each rumor. So, combining them, enables a holistic approach to the analysis of the rumor diffusion process in space and time.

### Classification

We test our models for rumor detection and classification. First, we use the fitted epidemiological model parameters, to classify the rumors using the labels provided in the dataset. We consider two distinct classification tasks. One where we perform a multi-class classification predicting one of the four labels: True rumor, False rumor, Unverified rumor and Non-rumor. We then perform a binary classification, where we only consider the True and False rumors. To train our classifiers we use a Gradient Boosting Trees algorithm using the fitted model parameters as features and performing a very light hyper parameter tuning. Regarding both the graph-based features and the combined feature set containing graph and epidemiological features, we again employ Gradient Boosting Trees, because both graph and epidemiological features are easily tabulated and the algorithm works well for numerical and categorical values, as in our case. The Gradient Boosting Trees algorithm allows for the sequential connection of individual decision trees and requires very little data pre-processing; thus, it fits very well the particular data of the rumor diffusion problem.

We split the dataset 75/25 as a training and test set; this is typical in the prior work that uses these datasets. To evaluate the performance of the classifiers, we measure their accuracy, precision and recall on both classification tasks.

Next, we test whether the SEIZ model can detect false rumors by using a single ratio RSI of the fitted parameters as suggested by Jin et al. (2013). Their work suggests that larger ratios correspond to real news propagation, while smaller values to fake news spreading. The RSI value is a combination of the SEIZ fitted parameters for each rumor and we derive it in Eq. 9, where p, β, l, b, ρ and ε are the SEIZ fitted parameters:

(9) $$\begin{array}{r} R_{SI}\;=\;\frac{\left(1\;-\;p\right)\beta \;+\;\left(1\;-\;l\right)b}{\rho \;+\;\epsilon } \end{array}$$

**Figure 4:**
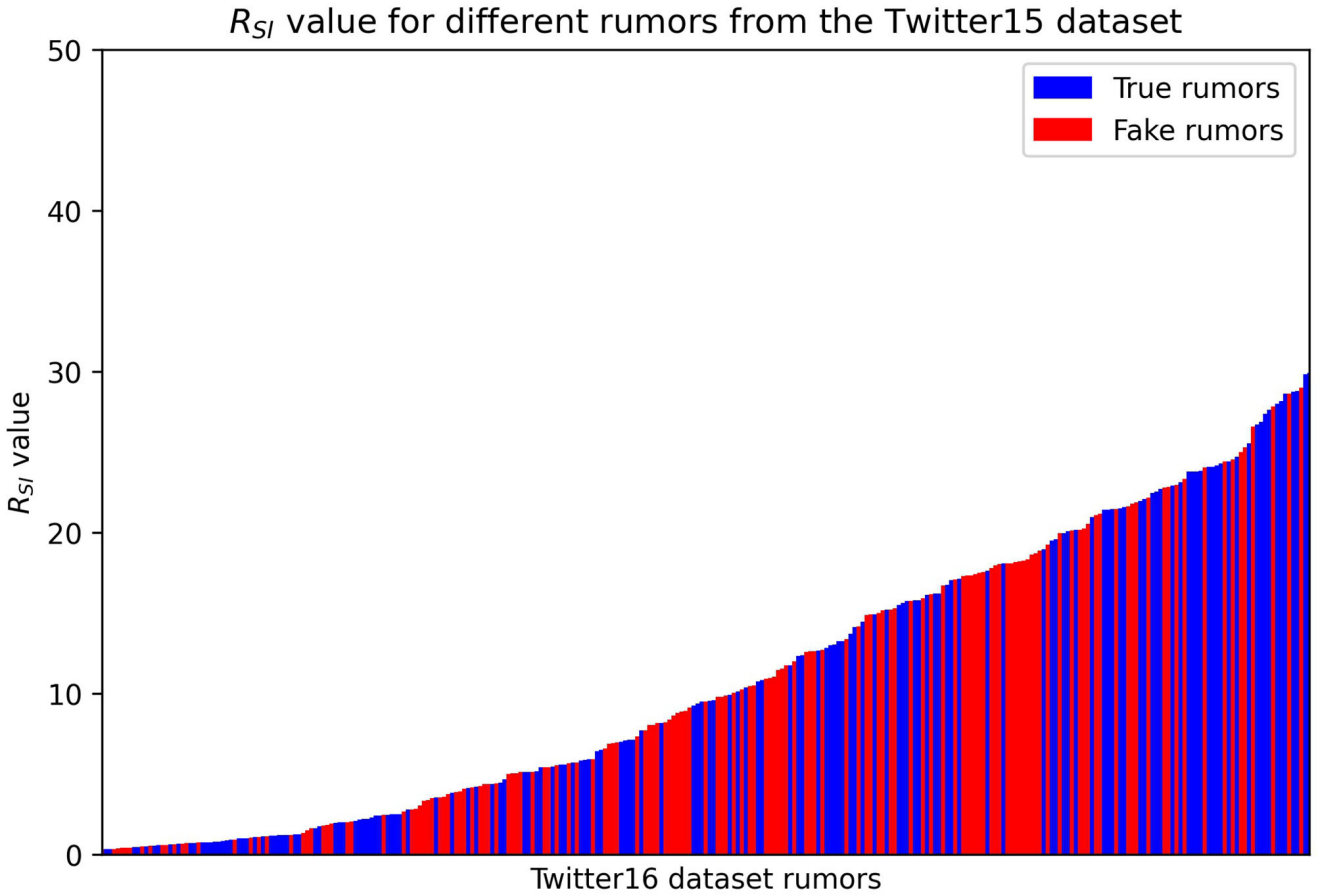
RSI values of different rumors. Red = False rumors, Blue = True rumors.

In Figure 4, we present the RSI values of different rumors; we denote the True rumors with blue color and the Fake ones with red.

One can easily observe that it is difficult to find a specific threshold value of RSI that would adequately classify the rumors. Based on the results we consider a more complex detection technique where we use a K-nearest neighbors algorithm, classifying a rumor based on its “closest neighbors.” We test two different values for the number of neighbors we consider for each rumor, five and 20 neighbors.

## Experiments and Evaluation

In this section we present our experiments, including the necessary pre-processing, and our evaluation results. We present the results of rumor modeling and classification using epidemiological features, graph-based features as well as using their combination. Finally, we compare our method with other similar existing approaches to misinformation diffusion.

### Dataset and Data Pre-Processing

We work with two well-known publicly available datasets, Twitter15 and Twitter16 (Ma et al., 2017). The datasets describe the diffusion trees of various rumors on Twitter. More specifically, they provide temporal information about every retweet of an original tweet containing a specific rumor posted by a single root user at *t* = 0.

An important limitation we should consider is that every rumor included in the datasets has a single root, meaning that we only have information about how the rumor propagated after a single user shared it on Twitter. In reality we would expect that rumors would have multiple users sharing them independently thus creating multiple independent parallel propagation trees.

We use the above mentioned Twitter15 and Twitter16, since, although other Twitter datasets may be publicly available, most of them do not publish the actual propagation information and instead provide tweets and users unique identifiers (IDs) that can be used to reconstruct the actual propagation tree using the Twitter API. This makes it very difficult to reproduce the original propagation sequences as significant numbers of users delete their own tweets and some even their accounts.

The datasets consist of 1,490 and 818 rumor propagation trees, respectively. The rumors themselves are labeled as True rumors, False rumors, Unverified rumors or Non-rumor events (Ma et al., 2017). True, False and Unverified labels refer to tweets containing unsubstantiated claims that could not be verified at the time of posting. Tweets that eventually get verified are labeled as True, tweets proved to contain fake claims are labeled as False, while tweets that contain information that can be neither confirmed nor disproved are labeled as Unverified. Finally, tweets that contain legitimate, fact-based information are considered Non-rumors. Table 1 summarizes key statistics of the dataset (Ma et al., 2017).

**Table 1:** Statistics of Twitter15 and Twitter16 datasets (Ma et al., 2017).

**Statistic**	**Twitter15**	**Twitter16**
Number of users	276,663	173,487
Number of tweets	1,490	818
Average time length/tree	1,337 h	848 h
Average posts/tree	223	251
Max posts/tree	1,768	2,765
Minimum posts / tree	55	81

We also perform some minimal data cleaning because we identified some minor inconsistencies in the dataset. Specifically, we noticed that some propagation trees contained negative timestamps and did not uniformly designate the root user who first tweeted the rumor. To correct these inconsistencies, we padded all timestamps in the affected propagation trees to make them positive and rearranged the trees so that root users appear in all rumors consistently.

### Epidemiological Model Results

Table 2 presents the average Root Mean Square Error (RMSE) for the different time limits for Twitter15 and Twitter16 datasets (lower values denote better fitting). We observe that the SI model, despite its simplicity, fits the data very well, while it marginally outperforms the more complex SEIZ model on the 240 h time limit experiment. Despite that, the SEIZ model performs better on all other experimental setups, especially on the early detection tasks where the diffusion is more rapid.

**Table 2:** Twitter15 and Twitter16 fitting error (RMSE).

	**Twitter15**		**Twitter16**	
	**SI**	**SEIZ**	**SI**	**SEIZ**
240 h	19.55	21.592	21.38	25.578
120 h	24.69	24.41	26.08	29.661
72 h	29.29	26.83	30.98	28.075
48 h	32.88	24.126	34.86	29.232
4 h	35.24	6.855	34.66	6.205

Table 3 presents the multiclass classification results for both models, where rumors are classified as True, False, Unverified or Non-Rumor in both Twitter15 and Twitter16 datasets. Table 4 presents the binary classification results, where rumors are classified as either True or False. The baseline accuracy for the multiclass classification is approximately 0.25 as 4 different classes are present and approximately 0.5 for the binary classification task as the dataset is almost perfectly balanced. The results indicate that both models perform almost the same at classifying the rumors. At the same time, the different time limits have low impact on performance. Interestingly, all models perform better on the Twitter16 dataset, indicating that Twitter16 is an easier dataset for our classifiers.

**Table 3:** Twitter15 and Twitter16 SI and SEIZ 4 class classification results.

	**Twitter15**				**Twitter16**			
	**Accuracy**	**F1 score**	**Precision**	**Recall**	**Accuracy**	**F1 score**	**Precision**	**Recall**
**SI**								
240 h	38.98	37.80	37.56	38.98	46.83	45.68	45.90	46.83
120 h	**42.47**	**42.05**	**41.86**	**42.47**	**49.76**	**49.47**	**49.91**	**49.76**
72 h	41.13	40.12	39.78	41.13	48.29	47.38	47.83	48.29
48 h	37.90	36.63	36.41	37.90	42.44	42.33	42.35	42.44
4 h	33.24	32.33	32.03	33.24	38.73	38.90	39.49	38.73
**SEIZ**								
240 h	**39.52**	**38.31**	**38.15**	**39.52**	46.34	45.49	46.25	46.34
120 h	37.10	36.67	36.50	37.10	**47.80**	**47.98**	**49.49**	**47.80**
72 h	36.56	35.64	35.51	36.56	45.37	45.61	46.16	45.37
48 h	37.90	36.98	36.85	37.90	40.49	40.26	40.47	40.49
4 h	36.76	35.62	35.46	36.76	36.76	36.61	36.84	36.76

*The best performing experimental setups are provided in bold*.

**Table 4:** Twitter15 and Twitter16 SI and SEIZ binary classification results.

	**Twitter15**				**Twitter16**			
	**Accuracy**	**F1**	**Precision**	**Recall**	**Accuracy**	**F1 score**	**Precision**	**Recall**
**SI**								
240 h	54.59	54.57	54.49	54.59	63.11	63.07	63.21	63.11
120 h	50.27	50.25	50.28	50.27	64.08	64.07	64.08	64.08
72 h	52.43	52.43	52.43	52.43	66.02	66.00	66.09	66.02
48 h	**54.59**	**54.57**	**54.59**	**54.59**	66.02	66.02	66.03	66.02
4 h	50.00	49.98	50.14	50.00	**67.96**	**67.69**	**68.47**	**67.96**
**SEIZ**								
240 h	51.89	51.86	51.91	51.89	60.19	60.15	60.21	60.19
120 h	49.19	49.11	49.21	49.19	63.11	63.09	63.11	63.11
72 h	52.43	52.42	52.45	52.43	65.05	65.01	65.17	65.05
48 h	54.59	54.45	54.62	54.89	**68.93**	**68.91**	**68.95**	**68.93**
4 h	**54.89**	**54.88**	**55.03**	**54.89**	66.02	65.98	66.05	66.02

*The best performing experimental setups are provided in bold*.

Table 5 presents binary classification results (True or False), using a nearest neighbor algorithm and considering the 5 and 20 nearest neighbors. For this classification, we use the fitted SEIZ parameters, combining them in a single RSI value for every rumor. Despite the simplicity of the technique, we get very similar results as we get from using the more complex Gradient Boosting algorithm. Thus, we confirm that the RSI values can be used (to a certain extent) to detect fake rumors as suggested by Jin et al. (2013). However, no single threshold value that splits the data efficiently was identified in our experiments.

**Table 5:** RSI classification results for Twitter15 and Twitter16 datasets.

	**5 Nearest Neighbors**				**20 Nearest Neighbors**			
	**Accuracy**	**F1 score**	**Precision**	**Recall**	**Accuracy**	**F1 score**	**Precision**	**Recall**
**RSI for Twitter15**								
240 hours	**51.35**	**51.01**	**51.44**	**51.35**	**54.05**	**53.91**	**54.14**	**54.05**
120 hours	48.62	48.62	48.62	48.62	49.19	49.11	49.21	49.19
72 hours	49.24	49.24	49.24	49.24	52.97	52.92	53.01	52.97
48 hours	48.61	48.61	48.61	48.61	49.73	49.62	49.75	49.73
4 hours	50.57	50.57	50.57	50.57	50.00	48.36	50.73	50.00
**RSI for Twitter16**								
240 hours	**64.08**	**64.08**	**64.09**	**64.08**	58.25	57.16	59.40	58.25
120 hours	52.43	52.43	52.50	52.43	55.34	54.31	56.05	55.34
72 hours	54.37	54.32	54.36	54.37	54.37	54.06	54.59	54.37
48 hours	61.17	61.05	61.36	61.17	**63.11**	**62.64**	**63.94**	**63.11**
4 hours	51.46	51.43	51.44	51.46	50.49	48.64	50.36	50.49

*The best performing experimental setups are provided in bold*.

Although epidemiological models yield reasonable results, all models fail to adequately classify the rumors, especially in the Twitter15 dataset. The limitations are mainly two: (i) epidemiological models fail to account for the structural components of the diffusion tree and (ii) machine learning models are trained on fitted parameters -instead of the real data- and the models ultimately produce underperforming detection results despite the robust fitting.

### Graph Model Results

Graph-based modeling is exploited to capture the structural components of the diffusion mechanism, in order to overcome the limitations of the epidemiological models in rumor classification.

**Figure 5:**
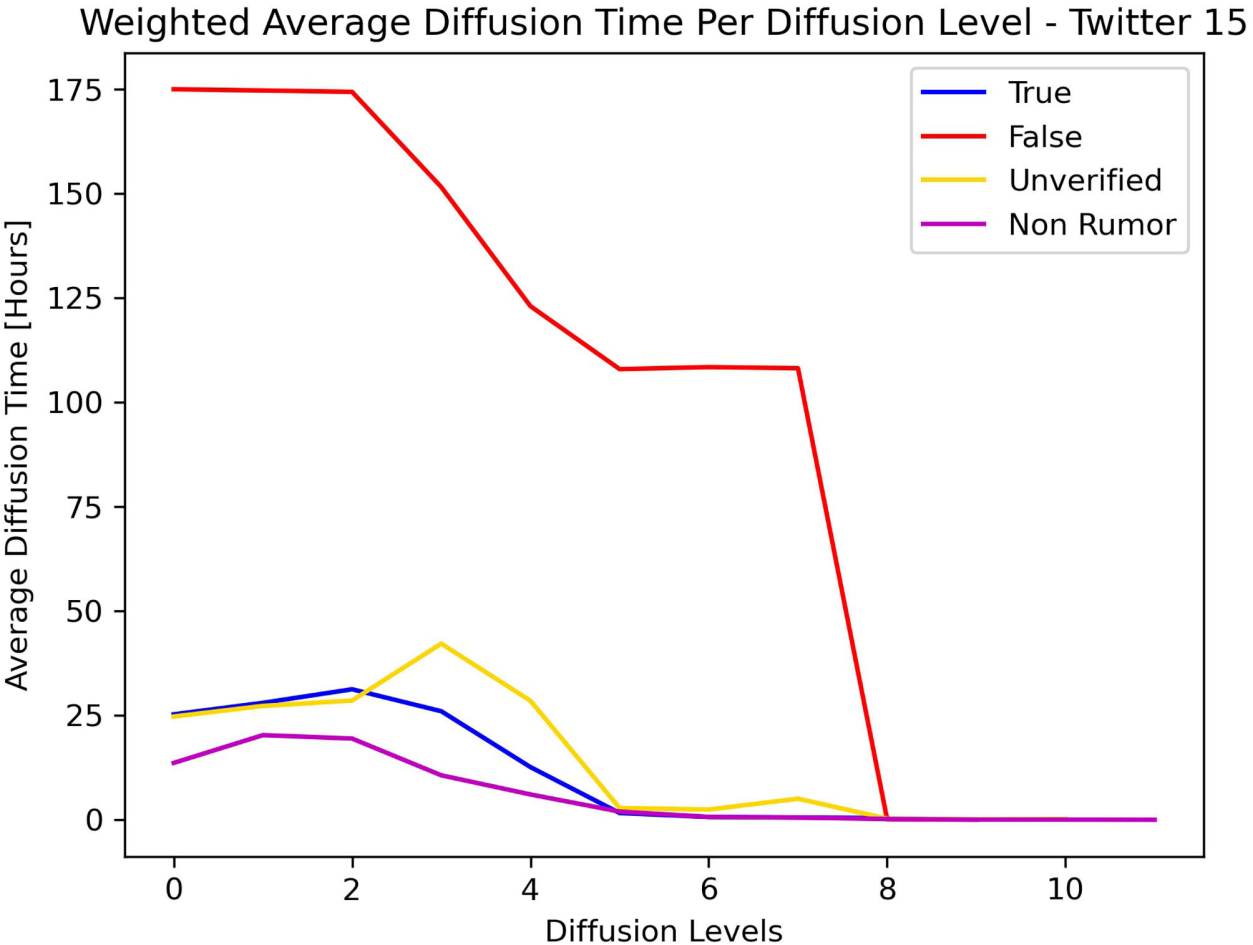
Weighted average diffusion time per diffusion level—Twitter15.

**Figure 6:**
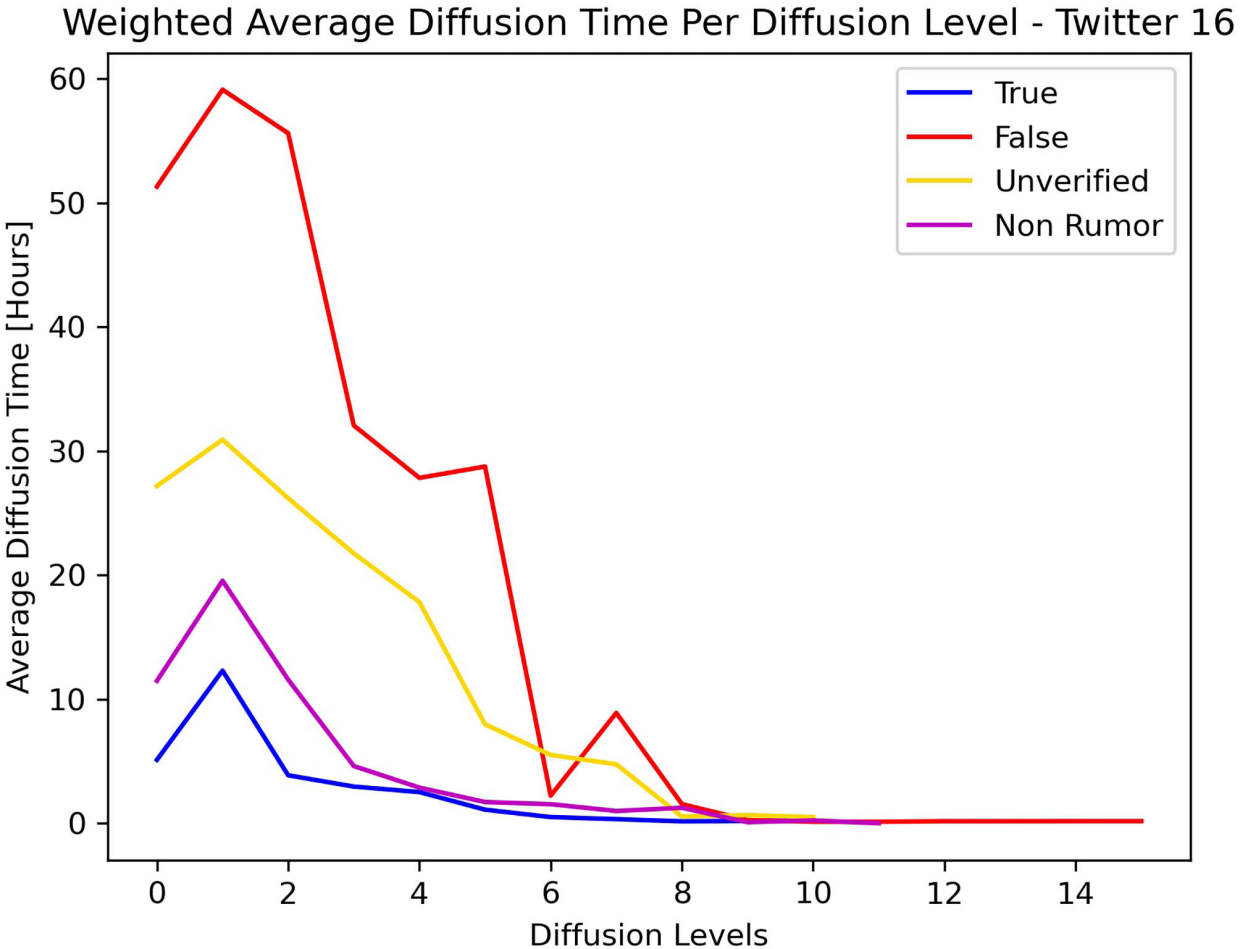
Weighted average diffusion time per diffusion level—Twitter16.

Figures 5, 6 show the average diffusion time per level (Eq. 9) for the Twitter15 and Twitter16 datasets, respectively. The calculation of the average diffusion time incorporates information about the population of users of each cascade in the form of maximum hops from the root user. We identify one similarity among the two datasets: the cascades of False label propagate at a much slower rate than the cascades of any other label (True, Non-Rumor, Unverified) in the two initial levels of diffusion. We exploit this observation to establish an early detection mechanism.

Using the graph-based features, we perform binary and 4-class classification. For these two classification schemes, we employ a Gradient Boosting algorithm (as described in section classification) and we present the results for the binary and 4-class classifications in Tables 6, 7, respectively. The feature set regarding the graph-based features is denoted as “Graph + < X Hours ADT,” where X denotes the upper time threshold for the Average Diffusion Time calculation for the particular feature set. By producing results based on an upper time threshold for the Average Diffusion Time, we can evaluate whether graph-based features may be used efficiently for early misinformation detection. Regarding the Twitter15 dataset, we observe that for both binary and 4-class classifications, the best accuracy is achieved in the under-24-h time threshold (60.22% and 40.48%, respectively). However, for the Twitter16 dataset, the best accuracy is achieved using the full extent time for the binary classification (82.52%) and the under-48-h time threshold for the 4-class classification (60%). The Twitter15 dataset is much larger and contains more users and tweets than Twitter16, which seems to be an easier dataset for our classifiers. Given the observed discrepancy in the results between the two datasets, we cannot safely conclude on whether using solely graph-based features is sufficient for an early detection mechanism.

**Table 6:** Twitter15 and 16 Graph-based and combined feature sets—binary classification results with labels = {true, false}.

	**Graph-based feature set binary classification**							
	**Twitter15**				**Twitter16**			
**Feature Set**	**Accuracy**	**F1**	**Precision**	**Recall**	**Accuracy**	**F1**	**Precision**	**Recall**
Graph + <4 h ADT	58.60	59.69	58.16	61.29	75.73	76.19	72.73	80.00
Graph + <24 h ADT	60.22	59.34	60.67	58.06	77.67	77.67	75.47	80.00
Graph + <48 h ADT	57.53	56.83	57.78	55.91	75.73	77.06	71.19	84.00
Graph + <72 h ADT	**60.22**	**61.46**	**59.60**	**63.44**	77.50	78.50	73.68	84.00
Graph + Total ADT	57.53	58.64	57.14	60.22	**82.52**	**82.69**	**79.63**	**86.00**
Graph + SEIZ feature set binary classification								
Graph + <4 h ADT + SEIZ	62.24	56.98	59.76	54.44	73.79	74.29	70.91	78.00
Graph + <48 h ADT + SEIZ	**63.13**	**61.38**	**58.00**	**65.17**	75.73	76.64	71.93	82.00
Graph + <72 h ADT + SEIZ	58.08	56.08	53.00	59.55	**77.67**	**78.10**	**74.55**	**82.00**

*The best performing experimental setups are provided in bold*.

**Table 7:** Twitter15 and 16 Graph-based and combined feature sets −4-class classification results.

	**Graph-based feature set 4-class classification**							
	**Twitter15**				**Twitter16**			
**Feature Set**	**Accuracy**	**F1**	**Precision**	**Recall**	**Accuracy**	**F1**	**Precision**	**Recall**
Graph + <4 h ADT	37.27	37.66	38.54	37.27	51.71	51.80	52.60	51.71
Graph + <24 h ADT	**40.48**	**40.19**	**40.19**	**40.48**	56.10	56.01	57.34	56.10
Graph + <48 h ADT	39.68	39.20	38.95	39.68	**60.00**	**59.42**	**59.95**	**60.00**
Graph + <72 h ADT	37.53	37.09	36.99	37.53	59.02	58.75	59.10	59.02
Graph + Total ADT	38.34	38.41	39.02	38.34	52.20	51.86	51.75	52.20
Graph + SEIZ feature set 4-class classification								
Graph + <4 h ADT + SEIZ	**46.54**	**44.74**	**44.72**	**46.54**	56.91	55.88	56.00	56.91
Graph + <48 h ADT + SEIZ	39.34	37.47	36.51	39.34	**62.35**	**61.42**	**61.88**	**62.35**
Graph + <72 h ADT + SEIZ	42.15	40.50	39.93	42.15	52.23	52.48	53.51	52.23

*The best performing experimental setups are provided in bold*.

### Combining Graph and Epidemiological Models

Since no correlation was found between the epidemiological and the graph-based features (as described in section Graph and Epidemiological Models Combination), we merge the two feature sets and we present the classification results for this combined feature set. The merging the two feature sets enables us to capture both temporal (epidemiological) and structural (graph) components of the rumor diffusion process. We employ a Gradient Boosting algorithm and we present the results for the binary and 4-class classification in Tables 6, 7, respectively. In both tables, we provide the results for the graph-based feature set as well as for the merged graph and SEIZ feature set, in order to enable a clear comparison. Following the same notation as before, we present the merged feature set, which is denoted as “Graph + < X Hours ADT + SEIZ,” where SEIZ refers to the epidemiological feature set and X denotes the upper time threshold for the Average Diffusion Time.

Clearly, in both binary and 4-class classification, the results are better in absolute values when we employ the merged feature set, in contrast to the epidemiological or the graph based ones independently. Furthermore, the best accuracy is achieved at a lower time threshold when employing the merged features. Given these two results, we conclude that the combination of the epidemiological (temporal) and the graph (spatial) features enable higher accuracy for early detection as well as higher detection accuracy in absolute value.

### Comparison With Existing Methods

Table 8 presents the comparison of our solution with alternative state-of-the-art approaches to the misinformation diffusion problem for to the 4-class classification results.

**Table 8:** Baseline models classification results for Twitter15 and Twitter16 datasets.

	**Accuracy**	
	**Twitter15**	**Twitter16**
DTC (Castillo et al., 2011)	45.4	46.5
SVM-RBF (Yang et al., 2012)	31.8	32.1
SVM-TS (Ma et al., 2015)	54.4	57.4
DTR (Zhao et al., 2015)	40.9	41.4
GRU (Ma et al., 2016)	64.6	63.3
RFC (Kwon et al., 2017)	56.5	58.5
PTK (Ma et al., 2017)	75.0	73.2
RvNN (Ma et al., 2018)	72.3	73.7
PPC (Liu and Wu, 2018)	84.2	86.3
Our approach	46.5	62.4

It is important to note that, our approach is the only approach that is location-independent and language-independent, since it is based on spreading (epidemiological) and network (graph) characteristics and does not take into account any characteristics of the content. In contrast, all existing methods consider content characteristics to some extent. Thus, the effectiveness of our approach cannot be directly compared with the results in the literature; the comparison with existing results is biased due to the consideration of content characteristics by all the methods we compare to. All the baseline approaches from the literature which are included in Table 8 combine content, user characteristics, and/or propagation-based features; this enables alternative solutions for higher accuracy results than our approach.

The experimental results of the baseline models that appear in Table 8 are drawn from the literature (Yuan, 2019; Ke et al., 2020). As Table 8 shows, our approach achieves results that rank it in the top 7 approaches for Twitter15 and the top 5 for Twitter16, in regard to accuracy, relatively to the 10 alternatives included in Table 8.

Importantly, another common characteristic of existing approaches is the employment of user characteristics extracted from the Twitter API, which allows for a plethora of features. However, such an approach renders the solution platform-specific. The use of the Twitter API introduces issues, such as the deletion of accounts and the loss of the associated features over time. This can be defined as dataset aging, where the data collected at a given point in time become gradually irrelevant and/or non-reproducible. In the case of Twitter15 and Twitter16, several users and tweets are no longer available on Twitter itself, due to account suspensions and/or thread deletions. This, in turn, leads to considerable difficulty in the reproduction of results and to transferability studies. Clearly, reliance on the particular characteristics of Twitter users or specific tweets is severely impeded by dataset aging. Our approach is oblivious to and unaffected by dataset aging, because it examines only the behavioral aspects of diffusion in space and time and does not consider any other external parameters.

## Conclusions

We propose a readily deployable solution for rumor detection on social media. The proposed framework is based on the diffusion cascade of an input rumor and does not require any additional user characteristics. This enables the use of the framework for any online social media platform, in contrast to existing literature that focuses only on Twitter. Furthermore, the framework does not require complex pre-trained language models or other high complexity content-based solutions and, thus, is applicable to real life systems, with no architectural adjustments or additional overhead.

Our method extracts temporal and structural features, enabling classification based on those features. The temporal-based features are extracted by fitting an epidemiological model on the original information diffusion data, and the structural features are gathered from a graph modeling of the same propagation data. We used the publicly available rumor propagation datasets Twitter15 and Twitter16 to evaluate the method. Experiments for binary (True, False) and 4-class (True, Non-Rumor, False, and Unverified) classification lead to the result that graph-based features perform better than epidemiological ones, while the most accurate classification is achieved through the combination of both feature sets.

Our method is location-independent and language-independent, leading to an approach that can be applied without consideration of the rumor content, in contrast to existing approaches that take into account rumor content. Our method is scalable and easily adaptable to existing real life systems with no modifications or additional overhead. However, it requires further investigation, due to dataset limitations. For example, our evaluations are for rumor cascades that begin from individual users, whereas in real life, multiple users may post the same rumor independently. Our method can accommodate multiple user sources, but no real dataset contains such information. Importantly, our method may provide improved results in that case, since machine learning techniques provide higher precision for large datasets.

The limitations of existing datasets are leading our future work, which focuses on collection of additional rumor propagation datasets from different online social networks and collection of data about rumors spreading from multiple root users, creating parallel diffusion trees. These datasets will enable us to evaluate the effectiveness of epidemiological models further and to evaluate graph-based features in rumor modeling and detection as well as overcoming some of the limitations of the Twitter15 and Twitter16 datasets.

## Data Availability Statement

Publicly available datasets were analyzed in this study. This data can be found here: https://www.dropbox.com/s/7ewzdrbelpmrnxu/rumdetect2017.zip?dl=0.

## Author Contributions

All authors contributed to manuscript revision, read, and approved the submitted version.

## Conflict of Interest

The authors declare that the research was conducted in the absence of any commercial or financial relationships that could be construed as a potential conflict of interest.

## Publisher's Note

All claims expressed in this article are solely those of the authors and do not necessarily represent those of their affiliated organizations, or those of the publisher, the editors and the reviewers. Any product that may be evaluated in this article, or claim that may be made by its manufacturer, is not guaranteed or endorsed by the publisher.
